# Pancreatic Metastasis from Prostate Cancer

**DOI:** 10.1155/2010/826273

**Published:** 2010-05-23

**Authors:** Julian Jacob, Cyrus Chargari, Olivier Bauduceau, Maryse Fayolle, Bernard Ceccaldi, Frédéric Prat, Sylvestre Le Moulec, Lionel Vedrine

**Affiliations:** ^1^Radiation and Medical Oncology Unit, Hôpital d'Instruction des Armées Val-de-Grâce, 74 boulevard de Port-Royal, 75005 Paris, France; ^2^Department of Oncology, Gastroenterology and Hepatology, Centre Hospitalier Universitaire Cochin, 75014 Paris, France

## Abstract

The pancreas is an unusual location for metastases from other primary cancers. Rarely, pancreatic metastases from kidney or colorectal cancers have been reported. However, a variety of other cancers may also spread to the pancreas. We report an exceptional case of pancreatic metastasis from prostate cancer. Differences in management between primary and secondary pancreatic tumors make recognition of metastases to the pancreas an objective of first importance. Knowledge of unusual locations for metastatic spread will reduce diagnostic delay and lead to a timely delivery of an appropriate treatment.

## 1. Introduction

The pancreas is an unusual location for metastases from other primary cancers. Rarely, pancreatic metastases from kidney or colorectal cancers have been reported [1]. However, a variety of other cancers may also spread to the pancreas. We report an exceptional case of pancreatic metastasis from prostate cancer.

## 2. Case Presentation

In August 2009, a 70-year-old man presented in the Emergency Unit with an acute cholestasis and decrease in general health status. He had a previous history of hormone refractory, high-grade prostate adenocarcinoma with bone metastases as first presentation (May 2007). The disease had progressed after endocrine therapy, then after first-line chemotherapy with docetaxel. The patient was currently treated with diethylstilbestrol. Physical examination showed marked icterus with no other clinical abnormalities. The liver was not palpable. Biological analysis revealed an important elevation of bilirubin (total bilirubin: 336 *μ*mol/L, conjugated bilirubin: 171 *μ*mol/L) and alkaline phosphatase (1.5 N). Gamma-glutamyl transferase remained within the normal range. Serum Prostatic-Specific Antigen (PSA) remained unchanged (259 ng/mL versus 283 ng/mL one month earlier). Abdominal echography and computed tomography showed a dilatation of intrahepatic bile duct, but no obvious tumor of the pancreas. The common bile duct was also dilated until pancreatic head, but no tumoral or lithiasic obstruction could be evidenced. There was no ascites. An MRI was performed, showing a heterogeneous increase of volume of the pancreas head so as hypertrophic retro-peritoneal nodes. The ultrasound endoscopy demonstrated a heterogenic mass that infiltrated the pancreatic head. 

 Endosonographic fine-needle biopsy and endoscopic retrograde cholangiopancreatography (ERCP) were performed. Histological examination of this infiltration revealed the presence of a metastasis from an undifferentiated carcinoma, with positive immunohistochemical staining for PSA, supporting the diagnosis of a pancreatic metastasis from a metastatic prostate adenocarcinoma. Due to poor performance status, symptomatic treatment only was decided. Unfortunately, the patient died in October 2009 from disease progression.

## 3. Discussion

The main site of metastasis in prostatic adenocarcinoma is the bone. Most atypical prostate carcinoma metastases are encountered in the presence of known advanced disease. Rarely, unusual metastases can be the only sign of distant spread or a presenting feature of cancer [2]. Pancreas may constitute a metastatic site for other primary tumors, such as kidney, colon, stomach, melanoma, or ovarian [1]. To our knowledge, only two cases of pancreatic metastasis from prostate cancer have been previously reported [3, 4]. This suggests that pancreatic tumors in patients with a history of nonpancreatic malignancy should be always considered to be a potential metastatic lesion at an unusual site. If feasible, pathological confirmation should be performed because pancreatic metastases may clinically or radiologically mimic a pancreatic primary tumour. Although the differential diagnosis between a primary pancreatic cancer and metastases of other adenocarcinomas may be complex, using common pathological and immunohistochemical techniques may provide relevant information. In selected patients, surgical extirpation of isolated metastases to the pancreas from various primary tumors may potentially improve outcome [5].

## 4. Conclusion

Differences in management between primary and secondary pancreatic tumors make recognition of metastases to the pancreas an objective of first importance [1]. Knowledge of unusual locations for metastatic spread will reduce diagnostic delay and lead to a timely delivery of an appropriate treatment.
